# Transforming researchers into writers through a series of semi-structured writing retreats: a mixed methods study

**DOI:** 10.1186/s12912-024-02261-9

**Published:** 2024-09-02

**Authors:** James Bonnamy, Lyndal Bugeja, Julia Morphet, Philip L. Russo, Gabrielle Brand

**Affiliations:** 1 School of Nursing and Midwifery, Sub-Faculty of Health Sciences, Faculty of Medicine, Nursing and Health Sciences, Monash University, 47-49 Moorooduc Highway, Frankston, VIC 3199 Australia; 2 Department of Forensic Medicine, Monash University, 553 St Kilda Road, Melbourne, VIC 3004 Australia; 3 Cabrini Research, Cabrini Health, 154 Wattletree Road, Malvern, VIC 3144 Australia; 4 Nursing and Midwifery, Avondale University, 582 Freemans Drive, Cooranbong, NSW 2265 Australia

**Keywords:** Semi-structured, Researcher development, Research outputs, Writing for publication, Writing retreat, Writing workshop

## Abstract

**Aim:**

To evaluate the experience and effectiveness of six semi-structured writing retreats on research publication quantity and quality for nursing and midwifery academics and research students.

**Background:**

Research publications are necessary to develop a track record to gain competitive funding and for promotion. Publications also improve the standing of universities because their performance is measured in-part by research outputs. However, there are challenges to writing for publication, especially for new nursing and midwifery academics and research students. Therefore, four of the authors initiated semi-structured writing retreats to support nursing and midwifery academics and research students to overcome these challenges.

**Methods:**

A mixed methods exploratory sequential design consisting of two distinct phases and data collection methods. In phase one, an online evaluation was administered to collect participant experiences which were then analysed using content analysis. In phase two, data about the quantity and quality of publications arising from each retreat was collected, and descriptive statistics performed.

**Results:**

A total of 70 participants responded to the online evaluation. Qualitative analysis of their responses demonstrated that the writing retreats were highly valued as they offered a collaborative environment with dedicated time to focus on writing for publication. Quantitative analysis identified 81 publications were planned over the six writing retreats. Of these, 60 have been published, 5 are under review, 5 have not yet been submitted, and 11 were abandoned.

**Conclusions:**

Findings demonstrated that our six semi-structured writing retreats enabled and developed nursing and midwifery academics and research students writing for publication. Semi-structured writing retreats are a research investment that enabled preparation of high-quality publications by offering protected time to write, expert peer review and collaboration and networking opportunities.

## Introduction

Writing retreats provide an environment to engage in collaborative writing, which is especially important for new nursing and midwifery academics and research students to help demystify the process and grow their confidence. Writing retreats are often semi-structured, offering opportunities for distraction-free writing, expert peer feedback on manuscripts as well as researcher training opportunities. In a recent integrative review, several benefits of writing retreats which contributed to increased publication outputs were identified, including: protected time to write, support to develop academic writing skills, and collaboration and mentoring from expert peers [1].

## Background

Writing for publication is a key skill for academics and research students because high-quality research outputs are used in-part as a measure of academic success [2]. A research output track record is also a recognised metric of university performance. By publishing their research, nursing and midwifery academics and research students create and share new knowledge to improve healthcare and advance the nursing and midwifery professions. For academics, a growing publication record is necessary to gain competitive research funding, achieve promotion and attract research students. Despite these expectations, new academics often describe their professional identities as educators rather than researchers who must write and publish. This can create tension for new academics because of the publish or perish expectation [3] which has seen increasing pressure for individual academics to publish [4].

While conducting research is given priority, writing for publication may receive little attention, and consequently publications may be abandoned [4]. Without a clear understanding of the writing process or support to grow the necessary academic skills to write, the expectations of new academics and research students may be misaligned with the realities of writing for publication. New nursing and midwifery academics also describe being under considerable pressure to simultaneously gain higher degrees, design and deliver high-quality teaching, supervise research students, as well as undertake and publish research upon their transition, yet they may struggle to meet all of these expectations concurrently [5].

The research culture and environment of universities are influential predictors of individual academic and organisational research performance, measured in part by publications in high-quality journals [6]. Switching from a siloed and competitive writing culture to a social and collaborative one through co-writing can improve publication outputs [7]. Many scholars have therefore championed academic writing retreats or workshops to focus on researcher development to be able to successfully write for publication [8]. Through purposeful planning, writing retreats can be experienced as, and benefit from, communities of practice, where a shared group of people cohere through appropriated enterprise on a mutual goal. The core elements of community of practice that should be given careful attention, include: mutuality of engagement, identity of participation [9, 10] and legitimate peripheral participation [10] which are defined in Table 1.

**Table 1:** Core community of practice concepts relevant to successful writing retreats

Mutuality of engagement	“the ability to engage with other members and respond in kind to their actions, and thus the ability to establish relationships…” [9]
Identity of participation	“identity constituted through relations of participation” [9]
Legitimate peripheral participation	“concerns the process by which newcomers become part of a community of practice” [10]

To build the circumstances for a successful research environment and to grow nursing and midwifery academic and research student writing for publication capacity, four (GB, JM, LB & PLR) of the authors developed bi-annual, semi-structured writing retreats in 2019, with the intent of enculturating researcher identity, building strong collaborative relationships and ensuring participants have protected time to write up their research. All academics and research students in the school were eligible to apply to attend, and if successful, were supported with a scholarship which covered meals, venue hire, and for an off-campus retreat, accommodation at the venue. Scholarships were competitive and based on readiness to write up research findings. Participants were eligible to attend more than one writing retreat, but needed to demonstrate submission of the manuscript they were writing at the previous retreat and work on a new manuscript at the next retreat.

Each semi-structured writing retreat was held over three-days and two-nights in early July and December, outside of peak teaching periods. They have been held both on and off-campus, and once online in February 2021 due to the COVID-19 pandemic. The off-campus writing retreats are held at a picturesque resort, approximately 150kms from Melbourne in Victoria, Australia, nestled between wetlands and the pristine beaches formed by the Bass Strait, on the land of The Bunurong Peoples. Each writing retreat offered 14 h of dedicated writing time in blocks of 2–3 h across the three-days. Each day featured opt-in research training activities which were tailored to the experience and skill level of the participants, for example: choosing a journal for publication, nailing the abstract, tips and tricks for writing “mojo”, and bouncing back after reviewer rejection.

The aim of this study was to explore the experiences and effectiveness of a series of six semi-structured writing retreats on nursing and midwifery academic and research student writing for publication. Two questions guided this study: (1) What is the experience of nursing and midwifery academics and research students attending semi-structured writing retreats, and (2) What impact do a series of semi-structured writing retreats have on participants research outputs?

## Methods

### Design

The design was a mixed methods exploratory sequential design consisting of two distinct phases and data collection methods. This approach allowed us to explore how the experience of the semi-structured writing retreats developed participant writing for publication. In phase one, qualitative data were collected at the end of each retreat through an anonymous online survey that included Likert-scale questions and free-text responses. Only the free-text responses were analysed for this study. In phase two, quantitative data, including the number and quality of publications arising from each writing retreat were collected and analysed using descriptive statistics.

### Participants

Nursing and midwifery academics from Assistant Lecturer to Professor level and research students from Honours to PhD participated in the writing retreats. The participants had mixed publication experience, with some writing their first manuscript.

### Setting

This study was conducted at a single School of Nursing and Midwifery at an Australian university. During the period of this study, the school had over 70 academic staff, 25 professional staff and 2,600 students across undergraduate and postgraduate courses. The School typically has 31 students enrolled in their PhD program each year.

### Ethics approval and consent to participate

Retrospective ethics approval and a waiver of consent was obtained from the Monash University Human Research Ethics Committee (35531).

### Data collection

At the end of each writing retreat, participants were invited to complete an anonymous online evaluation via a Google Form. Completion of the evaluation was voluntary. The evaluation included Likert-type questions and open-ended answers to explain ratings. For example, a Likert-type question included ‘How would you rate your satisfaction with the writing block sessions?’ whilst an open-ended question included ‘How was the writing retreat beneficial for developing relationships/connections with other participants?’. Research outputs arising from each retreat were recorded by the research office. This data included participant role, output type, status of the publication (as of 19th February 2024), journal name, journal quartile ranking and impact factor, Altmetric score and Google Scholar citations. If a publication was abandoned the reason for abandonment, if available, was recorded. Prior to data analysis, data cleaning was undertaken by LB and JB.

### Data analysis

#### Qualitative analysis

In phase one, content analysis was undertaken “to provide knowledge and understanding of the phenomenon under study” through the evaluation free-text responses [11]. A deductive coding approach was applied, with a set of pre-defined deductive codes developed in a codebook. These codes were derived from concepts from Lave and Wenger’s communities of practice concepts and included: mutuality of engagement, identity of participation and legitimate peripheral participation [9, 12]. Credibility was established by two authors (JB & GB) blindly coding the data and then discussing any disagreements [13].

#### Quantitative analysis

In phase two, descriptive statistics were undertaken to measure the quantity and quality of publications arising from each retreat. The quality of the publications was measured through Clarivate Journal Citation Reports, specifically the impact factor and quartile ranking of the journal according to the year the publication was published. Journals are ranked into quartiles (Q1, Q2, Q3, and Q4) with Q1 being the top 25%. Quartile rankings are derived from journal impact factor data. This data can be influenced by a myriad of factors, including the type of publication, for example, review articles often attract more citations [14]. Altmetric scores and Google Scholar citations were also assessed to determine the reach of the publications.

#### Rigour

Rigour was established as described by Lincoln and Guba [15]. To maintain privacy, but also enable rigour in the breadth of participant data, each participant has been allocated an ID number based on the retreat they attended (numbered 1–6) and the order in which their feedback was received in the online evaluation, for example, the person who attended the first retreat and submitted their feedback third is labelled in the results as R1P3.

## Results

Over the six writing retreats a total of 112 participants attended: 94 academics from Assistant Lecturer to Professor level and 18 research students from Honours to PhD level. Participants were able to attend more than one writing retreat. Table 2 identifies the role and number of participants who have attended across the six writing retreats.

**Table 2:** Participants attending the writing retreats

Participant Role	Number^1^	Manuscripts Published^2^
Honours Student	**4**	**1**
Masters Student	**2**	**0**
PhD Candidate	**12**	**12**
Assistant Lecturer	**3**	**3**
Lecturer	**49**	**27**
Senior Lecturer	**28**	**11**
Associate Professor	**12**	**4**
Professor	**2**	**2**
**Total**	**112**	**60**

^1^ Participants could attend multiple writing retreats, therefore, the number of participants provided are not discrete participants, rather this data refers to the number of times a particular participant role title attended

^2^ To avoid double-counting, where multiple participants worked on a paper, the first author is counted only

### Qualitative findings

A total of 70 participants (62% of those eligible) submitted written comments to the online survey across five writing retreats: no data were collected for the February 2021 writing retreat due to the COVID-19 pandemic causing an abrupt end to the writing retreat.

### Mutuality of engagement

The ability to engage with other participants at the writing retreats and share both tangible and intangible efforts to establish relationships and achieve a common purpose was evident. While a few respondents preferred solitary writing, the majority were motivated to write when in the presence of others: “I felt motivated by other people writing” (R1P3). Sharing the same writing time and physical space meant that writers were “all on the same level” (R1P10) sharing the “time, energy and inspiration” (R1P10) of the retreat facilitators which encouraged writers to be “as productive as possible” (R2P2). There was a shared sense that by writing together it “enabled tasks to progress quicker than normal” (R3P13) because writers could “support each other when [they] had [writing] blocks” (R2P2). Writers having “similar issues and problems promoted sharing of knowledge” (R1P4) to sustain writing motivation throughout each retreat.

Sharing the writing experience meant that peer and expert feedback was readily available to enable and develop writing for publication: “overall support for all things academic writing was instantly at hand” (R3P14). Participants described having enthusiastic and experienced writing mentors as “invaluable” (R3P11) because they “received constructive feedback” (R1P7) and “pearls of wisdom” (R1P11) that provided the “perfect balance between support and challenge” (R1P16) to progress their writing. For novice writers, the “constant reassuring message[s]” (R1P16) encouraged them to continue writing during the retreat. For more experienced writers, they utilised the retreats to not only work on their own writing but to “[support] colleagues with developing their research/writing skills” (R3P15). Research students who attended appreciated dedicated time with their supervisors, thanking them in their responses for their “time and expertise” (R6P5) reviewing their writing and providing timely feedback.

The semi-structured and flexible approach was highly valued by participants. The “opt in, rather than compulsory” (R1P1) format of the research training activities meant that writers could continue writing or choose to take a break from writing and participate. One participant appreciated the “great balance between writing, interaction, [and] great teaching” (R3P9) whilst another valued “being given a freedom to choose where to write [and] that [they] could choose for how long to write” (R5P4). Not all writers chose to attend the research training sessions, but they acknowledged that whilst the sessions were “not necessary for [them], [they] can see value for others” (R6P10) highlighting appreciation for the shared learning experiences that were available. The semi-structured approach meant that writers experienced a “good mix of individual and group” (R5P6) writing time. The flexible approach also meant that participants were able to “spend time with people that [they] wouldn’t normally” (R1P14) and talk with “people about research but also about topics beyond research” (R1P17). The flexible and “social nature” (R2P2) of the retreats meant that writers were able to take time to hear about and learn from “what others are doing” (R2P2). Several participants identified that “having the ability to go elsewhere (to cabin, outside) was a good option” (R2P8) if they needed solitary thinking and writing time.

Five writing retreats were held face-to-face and one was held online. The contrasting experiences between the retreat formats highlighted how the physical environment influenced how participants were able to engage with and respond to each other. Overall, participants did not experience the online writing retreat as positively as the face-to-face retreats, finding that the retreat was “not [as] beneficial as [it was held] online” (R3P7). One participant of the online retreat missed the opportunity of seeing experienced academics and retreat facilitators “totally relaxed” (R1P15) which impacted on them feeling comfortable enough to approach them for feedback. Participants observed that the online retreat “had a big impact on developing relationships” (R3P5) and “developing connection” (R3P10) with other participants, demonstrating the value participants place on sharing the writing experience together. Another participant reflected that “no real connections [were] made” during the online retreat (R3P2). Distractions were more commonly identified during the online writing retreat with some participants finding it a “little tricker to switch off from work commitments” (R3P6).

### Identity of participation

The feeling of becoming a writer, and overcoming feelings of vulnerability, developed with time and participation in the writing blocks and research training opportunities. For most participants, prioritising writing during the retreat was evident: “went overtime with the blocks and at times just kept going in the moment” (R1P7). Some participants were conflicted about continuing to write or attending the opt-in research training sessions: “I would have liked to attend more but was conflicted as I had my writing mojo on, and wanted to keep writing while I was on a roll” (R1P17). One participant identified that the writing retreats “made [them] aware of focusing on writing without any other disturbances” (R1P21). Another participant “forced [themselves] to keep to the writing blocks and [were] actually very productive” (R3P8). Prioritising writing had many benefits for participants including feeling “like a weight had lifted off [their] shoulders” (R3P9) on the final afternoon when they identified their progress. There was a shared sense of “a lot of progress” towards writing up research results (R4P10). Most participants described “productive work” (R3P12) during the retreats including making “good progress on an aspect of [a] paper [they] had been stuck on for quite a few months” (R3P13). Progress made during the writing retreats meant that participants were “motivated to finish the paper” (R3P14) and submit shortly after the retreat. However, some participants felt disillusioned about their ability to continue writing on return to their usual academic responsibilities: “I just can never block out time – even with the suggestions given – I honestly don’t have the time” (R3P11). Another participant felt concerned that writing progress would slow “due to [their] teaching commitments” (R5P2).

Many of the participants were able to recognise the progress they made, which contributed to forming their identity as a writer: “we all feel vulnerable when it comes to writing…[but] it built my confidence” (R2P9). Another participant stated “I feel like my research brain has been challenged but has also grown, which is great” (R1P18). Developing an identity as a writer was seen in quotes like: “would not have thought [it] was possible” (R1P15) and by overcoming “the first major hump of getting something on paper” (R5P11). By the end of each retreat, many participants were about to “complete draft 1, which is epic” (R1P1).

### Legitimate peripheral participation

Sanctioning writing time enabled newcomers, including graduate research students, and academics at Lecturer level to dedicate time to the activities of the community of practice. The writing retreats provided “protected time to write” (R1P17) which offered teaching-intensive academics a “different focus” from their usual work (R1P10) and “guilt free time to write” (R1P18). Sanctioned writing time away from the usual competing responsibilities of academia allowed a collective and “concentrated effort to publish” (R1P15). An important aspect to engaging in protected writing time was the “emphasis that the facilitators put on protecting our time” (R1P16) which was “genuinely sanctioned” (R2P5) giving them a “licence to focus on [our] manuscripts” (R2P5). Dedicated writing time to “immerse yourself into the writing” (R2P2) was described as “very rare” (R1P20) whilst “being given permission” (R3P11) to spend dedicated time writing meant that everyone was on “equal footing” (R5P11). One participant identified that the retreats “demonstrated how much I can write” (R5P13) without distractions by “allowing the other tasks to wait” (R5P13). Whilst mostly positive, not everyone found the writing time allocated suitable or productive, with some participants finding it difficult to “maintain momentum for 3 days straight” (R6P2) whilst others “struggled to write and [then became] despondent” (R2P7).

For retreat participants, “access to such experienced academics” (R1P1) was central to believing they could achieve writing success, especially for novice academics. There was a perceived “commitment to staff development” (R1P11) evident in “time to discuss writing” (R2P2) with “like minded people” (R2P8). One participant identified that they “did not have [the] knowledge” to “choose a journal [or] write a manuscript” (R3P10) prior to attending, but “learnt an incredible amount” (R3P10) about these aspects during the retreat, reaching a first draft of their manuscript by the end. The “opportunity to learn new skills” (R3P13) from “experts sharing their experience” (R3P14) supported participant development of writing for publication. Participants were able to “gain advice and then have time to focus and write” (R6P5) with one research student sharing how their supervisor encouraged them to attend and “provided support so that [they] could finish [their] draft” (R6P1).

Participants appreciated the indulgence of writing, with one participant savouring the “sacred time to write” (R1P13) whilst another enjoyed the “beautiful inspiring location” (R2P8). All participants were complimentary to those “who made it possible” (R3P7). The “exec [executive] support to switch off emails” (R3P5) and focus specifically on writing for publication was also valued. One participant described their gratitude at “an otherwise impossible opportunity to write” (R5P8) whilst another appreciated “quarantined time to write” (R6P3) that came with being “away from campus” (R6P3). The retreats were also described as a “fantastic initiative” (R6P10) to exist in the “research space” (R1P9) to focus on writing. Participants who attended “looked forward to next year” (R5P2) and further opportunities to apply for a scholarship to attend another writing retreat. Despite the reality that research and writing for publication are core to the role of an academic, participants repeatedly described their gratitude at the “opportunity…to be away from work” (R5P10) where they could focus on writing for publication.

### Quantitative results

Data on the quantity and quality of research outputs arising from each participant or team was included up until 19th February 2024.

### Quantity of publications

Each of the writing retreats has generated high-quality research outputs. A total of 81 publications over six writing retreats were planned. Of these, 60 had been published (74%), 5 were under review (6%), 5 had not yet been submitted (6%), and 11 were abandoned (13%). Of the 11 publications that were abandoned, three were planned from Masters research and two from PhD research. The remaining six abandoned publications were planned to be written by academics at Lecturer level, with two of these academics leaving the university shortly after attending their respective writing retreat. Of the four-remaining abandoned publications, reasons were not provided. A summary of the research output data is included in Table 3.

**Table 3:** Summary of research output data from six semi-structured writing retreats

Retreat Date	Published		Submitted		Writing Continues		Abandoned		Total
	*n*	%	*n*	%	*n*	%	*n*	%	*n*
07-2019	10	67	0	0	1	7	4	26	**15**
12-2019	12	70	1	6	0	0	4	24	**17**
07-2020	7	100	0	0	0	0	0	0	**7**
02-2021	11	84	1	8	0	0	1	8	**13**
12-2021	14	67	2	9	4	19	1	5	**21**
07-2022	6	75	1	12	0	0	1	12	**8**
**Total**	**60**	**74**	**5**	**6**	**5**	**6**	**11**	**13**	**81**

### Quality of publications

Of the 60 published research outputs, 43 (71%) were published in Q1 or Q2 journals, with a further 11 (18%) published in Q3 or Q4 journals, and the remaining six (10%) not ranked in Clarivate. For the 60 research outputs published, the median impact factor in Clarivate was 3.19 (IQR 2.38–3.91). The highest ranked research output, based on journal quartile ranking and impact factor, was a systematic review published in the Journal of Clinical Oncology (Q1; IF 44.54; measured in Clarivate) by an Assistant Lecturer following the July 2020 writing retreat.

The range of Altmetric scores for all of the 60 published research outputs was 0–67, with the highest score (67) being for a qualitative systematic review protocol on diagnostic overshadowing and severe mental health published in the journal JBI Evidence Synthesis in 2021. The range of Google Scholar citation scores for the 60 published research outputs was 0–33, with the highest cited article being a scoping review about the nurses’ role in antimicrobial stewardship published in the International Journal of Nursing Studies in 2021.

## Discussion

There are diverse approaches to enhancing writing for publication for academics described in the literature [16], with semi-structured writing retreats offering a balance of research training, dedicated writing time and socialisation which is of benefit to novice writers [17]. We envisaged that a series of semi-structured writing retreats could offer protected time to write high-quality publications for busy academic staff and novice researchers. By offering opt-in research training sessions tailored to the skill mix and experience of participants at each writing retreat, we created a supportive atmosphere where “inevitable stumblings” when writing for publication became opportunities for shared growth and development among participants [9]. This supportive and collegiate environment saw a sustained decrease in the number of abandoned publications among graduate research students and academics at Lecturer level after the second writing retreat in 2019. Allocation of experienced mentors, regular peer-review of drafts, and targeted training sessions were part of the suite of planned activities to support writers to achieve publication submission.

The research found that writing retreats are experienced as a form of community of practice, for like-minded people who come together to share an experience to achieve an objective, in this case, to focus on developing writing high-quality publications. *Mutual engagement* is fundamental to communities of practice – people will come together with a common goal or interest and build a foundation on which everyone can participate in meaningful practice [9]. Retreat participants could simultaneously complete drafts and provide and receive feedback on manuscripts, feeding on “common knowledge, energy and a commitment to shared understandings” [18]. Our participants felt motivated by writing in the presence of their colleagues or fellow students because they were all working towards publishing their research outputs. Our flexible approach meant that writing retreat participants could spend time strengthening their social connections which facilitated a sense of collective engagement in the writing process.

*Identity of participation* [9] is crucial in forming communities of practice, it is illustrated by retreat participants seeing themselves as writers by being actively and collectively engaged in writing. Prioritisation of writing was evident in the tension participants experienced when they described wanting to attend more opt-in research training sessions but felt conflicted as they were also focussed on quantifiable writing progress. Progress on manuscripts which had sat latent for months highlights that participants could be writers when they were encouraged and given space and opportunity to write in an environment that was purposefully free of usual academic work distractions.

Protected writing time offered *legitimate peripheral participation*, allowing participants the opportunity to engage in focused writing for publication. Everyone who attended the writing retreats was exposed to the full scope of writing for publication, including the challenges to writing faced by even the most experienced academics. Witnessing the challenges of senior academics was important for novice writers, because it demonstrated the vulnerability involved in writing for publication including the non-linear iterative nature of the writing process. By removing the constraints of everyday work, everyone was able to participate fully in the writing process, sharing the writing experience and providing constructive feedback to improve writing.

The quantity and quality of publications arising from the six writing retreats demonstrates success of this investment in researcher development. A total of 60 research outputs have been published, with 71% in Q1 or Q2 ranked journals. Increased research outputs are essential in the current climate where quantity and quality of research output is directly associated with funding opportunities, attraction of research students and university rankings [19].

Writing retreats provide space and time for dedicated writing, but also opportunities for shared learning, and commitment to changing practice, to not only legitimise, but encourage writing by academics as part of their everyday work, even when they return to their usual work. However, time and space for writing can be difficult to obtain and may not be universally experienced by academics. This study demonstrated that semi-structured writing retreats provided the necessary elements to build and sustain a culture where writing for publication is part of academics and research students core work.

### Strengths and limitations

Both strengths and limitations exist for this study. Unlike previous studies, a strength of this study is that it reports on longitudinal data from six retreats over three years, and therefore can demonstrate the success of this research investment over a period of time [8, 20, 21]. Another strength of this paper is that it describes the range in the quality of the research outputs, whereas many previous studies have only identified the number of outputs from each retreat [4, 22] or focused only on participant experiences [20, 23–25].

Limitations included missing data (participant experiences) from the February 2021 writing retreat which is common in retrospective studies [26]. Another limitation is the reliance on the manual collection of publication data with some outputs potentially not identified. To mitigate this limitation, two authors (JB & GB) independently cross-matched participant attendance against research output data with a third author (LB) checking any discrepancies. Finally, the qualitative phase of this study included free-text responses from an online survey. It has been argued that analysis of free-text responses in online surveys “rarely meets the bar for rigorous qualitative work” due to the superficiality of the responses [27], including typically being devoid of context, personal meaning, emotional and social nuances and multiple layers of description [27]. Despite this limitation, and acknowledging that it would be useful to interview participants to gain rich accounts of their experiences, we believe this study adds to our understanding of the effectiveness of academic writing retreats.

Conclusion.

This research contributes to our understanding of the effectiveness of semi-structured writing retreats on writing for publication for nursing and midwifery academics and research students, which is currently underreported in the literature. This study demonstrates that semi-structured writing retreats can develop nursing and midwifery academic and research student writing for publication. Writing for publication is essential to disseminate research findings, academic success and raise the profile of nursing and midwifery research, therefore we argue that semi-structured writing retreats should be prioritised as an investment in writing for publication, a core but underappreciated component of the research process. Finally, this research provides an effective structure for those considering establishing their own semi-structured writing retreats to support academic writing for publication.

## Data Availability

The datasets generated and/or analysed during the current study are not publicly available due ethics restrictions.
